# Investigating risk factors for medication errors during perioperative care: A retrospective cohort study

**DOI:** 10.1097/MD.0000000000038429

**Published:** 2024-05-31

**Authors:** Lu Mo, Zhongxun Wu

**Affiliations:** a Department of Operating Room, The Affiliated Hospital, Southwest Medical University, Luzhou City, Sichuan Province, China.

**Keywords:** medication errors, nursing care, perioperative care, retrospective cohort study, risk factors, tertiary hospital

## Abstract

Medication errors during perioperative care significantly compromise patient safety and the quality of outcomes. It is crucial to identify and understand the factors that contribute to these errors to develop effective, targeted interventions. This study aims to explore the risk factors associated with medication errors during perioperative care in a tertiary hospital setting, focusing on patient demographics, medication types, administration routes, and nursing care characteristics. A retrospective cohort study was conducted, encompassing adult patients who underwent surgical procedures from January 2020 to January 2023. Data on medication administration, patient demographics, and surgical details were extracted from electronic health records. Medication errors were classified based on the harm caused to the patients. Logistic regression analyses were employed to identify significant risk factors. The study included 1723 patients, with a balanced gender distribution. The median patient age was 53 years. Medication errors were significantly associated with patient age, the type of medication administered, and specific administration routes. Higher education levels and advanced professional titles among nursing staff were inversely related to the occurrence of medication errors. The presence of a dedicated anesthesia nurse significantly reduced the likelihood of errors. Patient age, medication type, administration route, nursing education level, and the involvement of specialized anesthesia nurses emerged as significant factors influencing the risk of medication errors in perioperative care. These findings underscore the need for targeted educational and procedural interventions to mitigate such errors, enhancing patient safety in surgical settings.

## 1. Introduction

Medication errors in perioperative care present a significant challenge to patient safety, with the potential to cause adverse outcomes and prolong hospital stays.^[1–3]^ Despite stringent protocols and safety measures, the intricate nature of surgical care and the high-pressure environment often lead to medication administration errors.^[4–6]^ The perioperative setting is especially vulnerable to these errors, owing to the involvement of multiple healthcare professionals and the necessity for swift decision-making.^[7–9]^

Previous studies have underscored the multifactorial nature of medication errors, highlighting factors ranging from patient characteristics and medication types to healthcare provider practices.^[10–12]^ Notably, the nurse’s role in medication administration during surgery is pivotal, with their educational background, experience, and the presence of dedicated anesthesia nurses being identified as crucial determinants of error rates.^[13,14]^ Furthermore, patient demographics, such as age and underlying conditions, have been shown to influence the likelihood of experiencing medication errors.^[15,16]^

Although these factors are recognized, significant gaps persist in our understanding of their interactions and impacts within the perioperative context. Furthermore, the constantly evolving landscape of surgical care, driven by advancements in medications and technology, demands ongoing reassessment of these risk factors.

This study seeks to address these gaps by thoroughly examining the risk factors for medication errors during perioperative care in a tertiary hospital setting. By analyzing various factors such as patient demographics, types of medication, methods of administration, and nursing care features, this research aims to offer insights that could lead to specific interventions to improve patient safety and the quality of care in surgical environments.

## 2. Methods

### 2.1. Study design and participant selection

In this retrospective cohort study, conducted in a tertiary healthcare setting from January 2020 to January 2023, we investigated the risk factors for medication errors during perioperative care, focusing on adults who underwent surgery. To be included, participants had to be 18 years or older and have all medication during surgery managed solely by nursing staff. This approach ensured consistency with the medication practicality and generic drug evaluation criteria set by the State Administration for Market Regulation. We excluded any records that were incomplete or lacked detailed documentation of perioperative medication administration. We also excluded cases where medication was administered by personnel other than nursing staff, such as anesthesiologists or surgical doctors. These strict inclusion and exclusion criteria allowed us to precisely assess the incidence and factors influencing medication errors in surgical care.

### 2.2. Data collection

Data were meticulously gathered from the hospital’s electronic health records. Variables collected included demographic details (gender, age), patient education level, residential location, medication types, administration routes, surgical procedure types, and nursing care characteristics, including nurse age, educational background, professional title, and the allocation of a dedicated anesthesia nurse. Medication errors were identified based on predefined criteria that included any deviation from the standard medication protocol.

### 2.3. Statistical analysis

Baseline characteristics were described using medians and interquartile ranges for continuous variables, and frequencies and percentages for categorical variables. Logistic regression analysis was employed for both univariate and multivariate models to determine the association between potential risk factors and the occurrence of medication errors. The results were expressed as odds ratios with 95% confidence intervals, and a *P* value of <.05 was deemed to indicate statistical significance. Statistical analyses were performed using IBM SPSS Statistics version 26.0.

## 3. Results

### 3.1. Baseline characteristics of the study population

Table 1 presents the baseline characteristics of the study population. The sample is evenly divided by gender, with 863 (50.1%) females and 860 (49.9%) males. The median age of patients is 53 years, with an interquartile range (IQR) from 37 to 68 years. When considering the education level of patients, a relatively even distribution is observed: 451 (26.2%) have primary education, 432 (25.1%) have middle school education, 426 (24.7%) have completed high school, and 414 (24%) have attained a college degree or higher. The population is almost equally split between rural and urban living areas, with 852 (49.4%) residing in rural areas and 871 (50.6%) in urban areas. Regarding the type of medication administered, analgesics were given to 434 (25.2%) of the patients, making them the most common medication type, followed by antibiotics at 485 (28.1%), anticoagulants at 404 (23.4%), and anesthetics at 400 (23.2%). The administration routes of these medications varied: micro-pump Intravenous (IV) injection was used for 288 (16.7%) of the cases, inhalation for 258 (15%), subcutaneous injection for 280 (16.3%), IV drip for 377 (21.9%), intramuscular injection for 248 (14.4%), and IV injection for 272 (15.8%). The types of surgery performed were predominantly open surgery, accounting for 1078 (62.6%) of the cases, while minimally invasive surgery was performed in 645 (37.4%) cases. The median age of the administering nurses was 36 years, with an IQR from 31 to 41 years. The educational levels among the nurses showed that 240 (13.9%) had technical secondary school education, 405 (23.5%) had junior college degrees, 509 (29.5%) were undergraduates, and 569 (33%) had a master’s degree or higher. The distribution of nurse titles revealed that 245 (14.2%) were nurses, 376 (21.8%) were nursing officers, 490 (28.4%) were senior nursing officers, and 612 (35.5%) were deputy chief nursing officers. A majority of the patients, 1057 (61.3%), were attended to by dedicated anesthesia nurses, whereas 666 (38.7%) were not.

**Table 1:** Baseline characteristics of the study population.

Characteristics	Overall
Gender, n (%)	
Female	863 (50.1%)
Male	860 (49.9%)
Patient age, median (IQR)	53 (37, 68)
Patient education level, n (%)	
Primary	451 (26.2%)
Middle	432 (25.1%)
High school	426 (24.7%)
College and above	414 (24%)
Patient living area, n (%)	
Rural	852 (49.4%)
Urban	871 (50.6%)
Medication type, n (%)	
Analgesics	434 (25.2%)
Antibiotics	485 (28.1%)
Anticoagulants	404 (23.4%)
Anesthetics	400 (23.2%)
Administration route, n (%)	
Micro-pump IV injection	288 (16.7%)
Inhalation	258 (15%)
Subcutaneous injection	280 (16.3%)
IV drip	377 (21.9%)
Intramuscular injection	248 (14.4%)
IV injection	272 (15.8%)
Surgery type, n (%)	
Open surgery	1078 (62.6%)
Minimally invasive surgery	645 (37.4%)
Nurse age, median (IQR)	36 (31, 41)
Nurse education level, n (%)	
Technical secondary school	240 (13.9%)
Junior college	405 (23.5%)
Undergraduate	509 (29.5%)
Master and above	569 (33%)
Nurse title, n (%)	
Nurse	245 (14.2%)
Nursing officer	376 (21.8%)
Senior nursing officer	490 (28.4%)
Deputy chief nursing officer	612 (35.5%)
Dedicated anesthesia nurse, n (%)	
Yes	1057 (61.3%)
No	666 (38.7%)

Micro-pump IV injection: Administration of medication through a micro-pump for intravenous injection.

IQR = interquartile range, IV = intravenous.

### 3.2. Comparison of characteristics between medication error and no medication error groups

Table 2 delves into the characteristics of patients and nurses, comparing those who experienced medication errors with those who did not, and assessing the statistical significance of differences between these 2 groups. The study involved 298 cases with medication errors and 1425 cases without. Gender distribution across both groups showed no significant difference (*P* = .355), with females representing 8.2% (142) of the medication error cases and 41.8% (721) of the no medication error cases, while males accounted for 9.1% (156) and 40.9% (704) respectively. A significant age difference was observed; the median age for the medication error group was 64.5 years (Interquartile Range [IQR]: 52–76.75), compared to 50 years (IQR: 34–65) for the no medication error group, marked by a *P* value of < .001. This indicates that older patients were more likely to experience medication errors.

**Table 2:** Comparison of characteristics between medication error and no medication error groups.

Characteristics	Medication error	No medication error	*P* value
N	298	1425	
Gender, n (%)			.355
Female	142 (8.2%)	721 (41.8%)	
Male	156 (9.1%)	704 (40.9%)	
Patient age, median (IQR)	64.5 (52, 76.75)	50 (34, 65)	<.001
Patient education level, n (%)			.194
Primary	81 (4.7%)	370 (21.5%)	
Middle	76 (4.4%)	356 (20.7%)	
High school	83 (4.8%)	343 (19.9%)	
College and above	58 (3.4%)	356 (20.7%)	
Patient living area, n (%)			.579
Rural	143 (8.3%)	709 (41.1%)	
Urban	155 (9%)	716 (41.6%)	
Medication type, n (%)			<.001
Analgesics	76 (4.4%)	358 (20.8%)	
Antibiotics	110 (6.4%)	375 (21.8%)	
Anticoagulants	54 (3.1%)	350 (20.3%)	
Anesthetics	58 (3.4%)	342 (19.8%)	
Administration route, n (%)			<.001
Micro-pump IV injection	51 (3%)	237 (13.8%)	
Inhalation	29 (1.7%)	229 (13.3%)	
Subcutaneous injection	26 (1.5%)	254 (14.7%)	
IV drip	100 (5.8%)	277 (16.1%)	
Intramuscular injection	34 (2%)	214 (12.4%)	
IV injection	58 (3.4%)	214 (12.4%)	
Surgery type, n (%)			<.001
Open surgery	100 (5.8%)	978 (56.8%)	
Minimally invasive surgery	198 (11.5%)	447 (25.9%)	
Nurse age, median (IQR)	45 (40, 50)	34 (29, 39)	<.001
Nurse education level, n (%)			<.001
Technical secondary school	119 (6.9%)	121 (7%)	
Junior college	88 (5.1%)	317 (18.4%)	
Undergraduate	58 (3.4%)	451 (26.2%)	
Master and above	33 (1.9%)	536 (31.1%)	
Nurse title, n (%)			<.001
Nurse	110 (6.4%)	135 (7.8%)	
Nursing officer	102 (5.9%)	274 (15.9%)	
Senior nursing officer	56 (3.3%)	434 (25.2%)	
Deputy chief nursing officer	30 (1.7%)	582 (33.8%)	
Dedicated anesthesia nurse, n (%)			<.001
Yes	96 (5.6%)	961 (55.8%)	
No	202 (11.7%)	464 (26.9%)	

*P* value: Statistical significance, with values <.05 indicating a significant difference between the Medication Error and No Medication Error groups.

IQR = interquartile range, IV = intravenous.

When examining patient education level, no statistically significant difference was found (*P* = .194), despite the distribution across different education levels. Similarly, the living area of patients (urban vs rural) showed no significant impact on medication errors (*P* = .579). Significant differences were found in the type of medication (*P* < .001) and the route of administration (*P* < .001). For instance, antibiotics were associated with medication errors at a rate of 6.4% (110) compared to 21.8% (375) in the no error group. Similarly, the administration route of IV drip was higher in the medication error group (5.8%, 100) compared to the no error group (16.1%, 277). The surgery type also showed significant differences; open surgery was less common in the medication error group (5.8%, 100) compared to the no medication error group (56.8%, 978), with a *P* value of < .001. Nurse characteristics revealed significant differences in age, with a median age of 45 (IQR: 40–50) in the medication error group versus 34 (IQR: 29–39) in the no error group (*P* < .001). Education levels and titles of nurses administering medication also differed significantly between the 2 groups (*P* < .001), suggesting that less experienced or lower-educated nurses may be more prone to medication errors. Finally, the presence of a dedicated anesthesia nurse showed a significant difference (*P* < .001), with 5.6% (96) in the medication error group and 55.8% (961) in the no error group, indicating that the involvement of specialized nurses might reduce the risk of medication errors.

### 3.3. Univariate and multivariate analysis of risk factors for medication errors

Table 3 presents both univariate and multivariate analyses to explore the risk factors associated with medication errors within the study population, totaling 1723 participants. The analysis provides insights into how various characteristics influence the likelihood of medication errors occurring. Gender does not show a significant association with medication errors in both analyses, with males having an odds ratio (OR) of 0.889 (95% CI: 0.692–1.141) in the univariate analysis, indicating no significant difference from females (*P* value = .355).

**Table 3:** Univariate and multivariate analysis of risk factors for medication errors.

Characteristics	Total (N)	Univariate analysis		Multivariate analysis	
		Odds ratio (95% CI)	*P* value	Odds ratio (95% CI)	*P* value
Gender	1723				
Female	863	Reference			
Male	860	0.889 (0.692–1.141)	.355		
Patient age	1723	0.949 (0.940–0.957)	**<.001**	0.946 (0.932–0.960)	**<.001**
Patient education level	1723				
Primary	451	Reference			
Middle	432	1.025 (0.726–1.448)	.886		
High school	426	0.905 (0.644–1.270)	.563		
College and above	414	1.344 (0.931–1.940)	.115		
Patient living area	1723				
Rural	852	Reference			
Urban	871	0.932 (0.726–1.196)	.579		
Medication type	1723				
Analgesics	434	Reference		Reference	
Antibiotics	485	0.724 (0.522–1.003)	.052	0.774 (0.438–1.369)	.379
Anticoagulants	404	1.376 (0.942–2.009)	.099	1.361 (0.707–2.621)	.357
Anesthetics	400	1.252 (0.863–1.817)	.237	0.872 (0.468–1.626)	.667
Administration route	1723				
Micro-pump IV injection	288	Reference		Reference	
Inhalation	258	1.699 (1.040–2.776)	**.034**	1.167 (0.510–2.667)	.715
Subcutaneous injection	280	2.102 (1.270–3.481)	**.004**	1.173 (0.533–2.581)	.691
IV drip	377	0.596 (0.408–0.871)	**.007**	0.631 (0.322–1.238)	.180
Intramuscular injection	248	1.354 (0.845–2.171)	.207	0.964 (0.434–2.140)	.928
IV injection	272	0.794 (0.522–1.207)	.281	0.670 (0.327–1.370)	.272
Surgery type	1723				
Open surgery	1078	Reference		Reference	
Minimally invasive surgery	645	0.231 (0.177–0.301)	**<.001**	0.305 (0.199–0.469)	**<.001**
Nurse age	1723	0.724 (0.697–0.751)	**<.001**	0.734 (0.698–0.772)	**<.001**
Nurse education level	1723				
Technical secondary school	240	Reference		Reference	
Junior college	405	3.543 (2.506–5.008)	**<.001**	2.658 (1.445–4.886)	**.002**
Undergraduate	509	7.647 (5.269–11.099)	**<.001**	5.109 (2.796–9.336)	**<.001**
Master and above	569	15.974 (10.359–24.633)	**<.001**	8.348 (4.395–15.859)	**<.001**
Nurse title	1723				
Nurse	245	Reference		Reference	
Nursing officer	376	2.189 (1.559–3.073)	**<.001**	2.183 (1.219–3.908)	**.009**
Senior nursing officer	490	6.315 (4.339–9.190)	**<.001**	5.633 (3.028–10.478)	**<.001**
Deputy chief nursing officer	612	15.807 (10.130–24.667)	**<.001**	12.475 (6.417–24.252)	**<.001**
Dedicated anesthesia nurse	1723				
Yes	1057	Reference		Reference	
No	666	0.229 (0.176–0.300)	**<.001**	0.291 (0.189–0.447)	**<.001**

Note:

• Odds Ratio (OR): Represents the odds that an outcome will occur given a particular exposure, compared to the odds of the outcome occurring in the absence of that exposure.

• 95% CI: 95% Confidence Interval, indicating the range within which the true value of the OR is expected to fall with 95% certainty.

• *P* value: Indicates the statistical significance of the observed differences or associations. A *P* value of <.05 is typically considered statistically significant.

• Reference: Used as a baseline category against which other categories are compared in the analysis.

Patient Age emerges as a significant factor; each additional year of age is associated with a decreased odds of medication errors, with an OR of 0.949 (95% CI: 0.940–0.957) in the univariate analysis and an OR of 0.946 (95% CI: 0.932–0.960) in the multivariate analysis, both showing *P* values of < .001. This suggests that older patients have a lower risk of experiencing medication errors. When examining Patient Education Level, no significant differences are noted across various education levels in comparison to the primary education level, indicating that patient education level does not significantly affect the likelihood of medication errors. Patient Living Area also does not significantly influence medication errors, with urban patients having an OR of 0.932 (95% CI: 0.726–1.196) compared to rural patients, and a *P* value of .579. Different Medication Types reveal interesting insights. While Analgesics serve as the reference category, Antibiotics show a borderline nonsignificant trend towards a reduced odds of medication errors in the univariate analysis (OR = 0.724, 95% CI: 0.522–1.003, *P* = .052), but this association does not remain significant in the multivariate analysis. Administration Routes display variability in risk; for example, Subcutaneous Injection significantly increases the odds of medication errors in the univariate analysis (OR = 2.102, 95% CI: 1.270–3.481, *P* = .004), though this significance does not persist in the multivariate analysis. Surgery Type is significantly associated with medication errors; Minimally Invasive Surgery substantially decreases the odds of errors compared to Open Surgery, with an OR of 0.231 (95% CI: 0.177–0.301) in the univariate analysis and an OR of 0.305 (95% CI: 0.199–0.469) in the multivariate analysis, both with *P* values of < .001. Nurse Age and Nurse Education Level both show a significant impact. Particularly, higher nurse education levels are associated with a reduced odds of medication errors, with the most pronounced effect seen in those with Master and Above degrees (OR = 15.974, 95% CI: 10.359–24.633 in univariate; OR = 8.348, 95% CI: 4.395–15.859 in multivariate, both *P* < .001). Nurse Title significantly influences medication errors, with Deputy Chief Nursing Officers having a markedly reduced odds of errors (OR = 15.807, 95% CI: 10.130–24.667 in univariate; OR = 12.475, 95% CI: 6.417–24.252 in multivariate, both *P* < .001). Having a Dedicated Anesthesia Nurse is significantly associated with a decreased odds of medication errors (OR = 0.229, 95% CI: 0.176–0.300 in univariate; OR = 0.291, 95% CI: 0.189–0.447 in multivariate, both *P* < .001), suggesting that specialized nursing care plays a crucial role in reducing medication errors. This analysis underscores the multifactorial nature of medication errors in hospital settings, highlighting the significant roles played by patient age, surgery type, nurse’s age, education, title, and the presence of dedicated anesthesia nursing care.

### 3.4. Risk factor impact and predictive accuracy in medication error analysis

**Figure 1.**
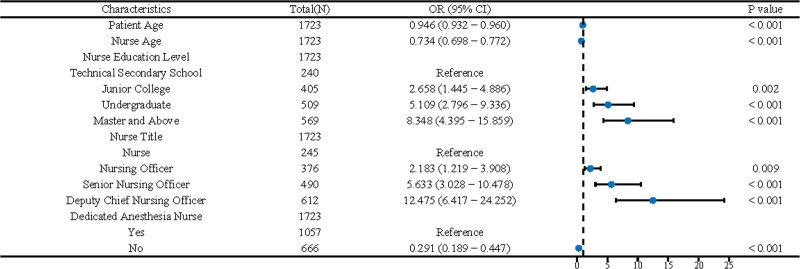
Odds ratios for medication error risk factors.

**Figure 2.**
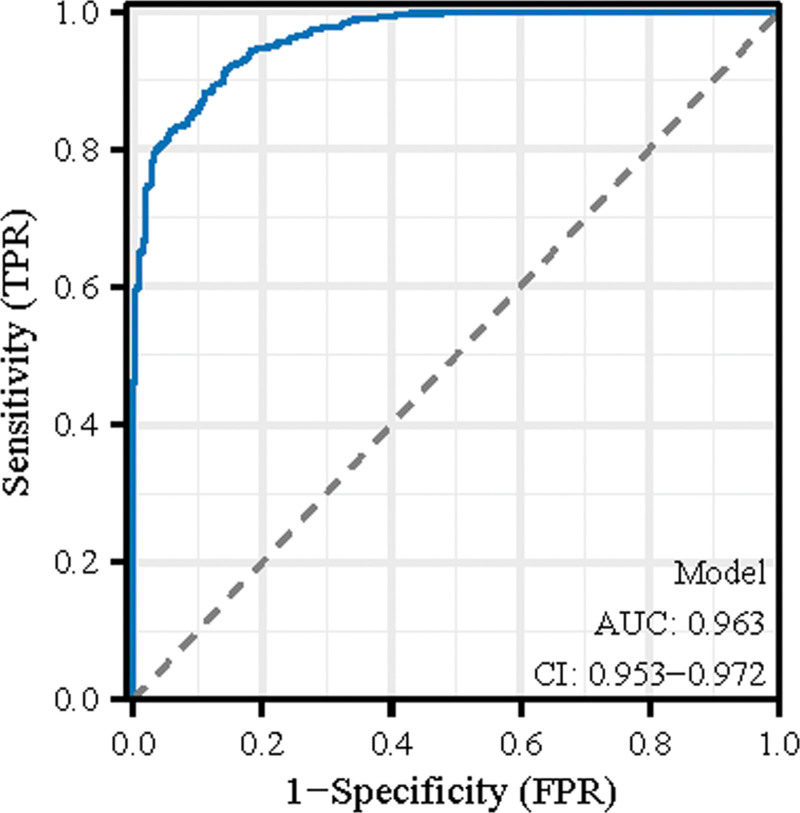
ROC curve for medication error prediction model.

Figure 1 serves as a forest plot that meticulously quantifies the influence of distinct risk factors on the incidence of medication errors. Displaying a range of variables, such as the ages of patients and nurses, the plot indicates that the level of nursing education and the presence of dedicated anesthesia nurses are pivotal factors. It reveals that increased education and advanced titles correlate inversely with the risk of medication errors, suggesting that a more educated nursing workforce is a critical component in mitigating this risk. Figure 2 showcases the ROC curve of our predictive model. The curve’s ascent towards the plot’s top-left, combined with an impressive area under the curve of 0.963, underscores the model’s precision in classifying cases correctly. This level of accuracy – echoed by the narrow confidence interval – validates the model’s efficacy in differentiating between instances with and without medication errors. Together, these figures substantiate the analytical model’s reliability and the significant role of specialized nursing care in the prevention of medication errors.

Legend:

•Blue dots represent the odds ratios for each risk factor.

•Horizontal lines indicate the 95% confidence intervals.

•Factors with intervals not crossing the dashed line (OR = 1) are statistically significant.

Legend:

•The curve traces the sensitivity (true positive rate) against 1-specificity (false positive rate).

•Area under the curve indicates the model’s accuracy, with a value of 0.963 signifying high predictive capability.

## 4. Discussion

This retrospective cohort study sheds light on the multifaceted nature of medication errors during perioperative care in a tertiary hospital setting, underscoring the critical role of patient demographics, medication types, and healthcare professional characteristics in influencing the incidence of these errors.

Consistent with previous research, our study found no significant association between patient gender and the occurrence of medication errors, reinforcing the findings of Nanji et al (2016)^[17]^ and Assiri et al (2019).^[18]^ However, the significant age difference observed between patients who experienced medication errors and those who did not suggests that older patients are at a higher risk, a finding that resonates with the observations of Assiri et al (2019) regarding the vulnerability of elderly patients to medication errors in community care settings.

Our analysis further highlighted the significant impact of the type of medication and route of administration on the likelihood of medication errors, echoing the concerns raised by Abbasi et al (2022) about the complexity of polypharmacy in perioperative care.^[19]^ The high incidence of errors associated with antibiotics and IV drip administration in our study underscores the need for targeted interventions to mitigate risks in these areas.

Moreover, the notable differences in nurse characteristics – specifically age, education level, and title – between the medication error and no error groups underscore the importance of healthcare professional training and experience in preventing medication errors, as suggested by the strategies for improvement highlighted in the literature.^[20]^ The protective effect of higher education levels and advanced nursing titles observed in our study suggests that enhancing educational programs and professional development opportunities for nursing staff could be a viable strategy to reduce medication errors in the perioperative setting.

The presence of a dedicated anesthesia nurse emerged as a significant protective factor against medication errors in our study, aligning with the findings of Wang et al (2023), who emphasized the importance of specialized nursing care in improving postoperative outcomes.^[21]^ This supports the notion that dedicated, specialized nursing roles can play a crucial role in enhancing patient safety during perioperative care.

Our study explores the complexities of medication errors during perioperative care within a tertiary healthcare setting but is subject to several limitations. The retrospective cohort design, although beneficial for analyzing existing records, cannot establish causality and may be biased by inconsistencies in documentation standards. Being confined to a single institution, the study’s findings may not apply universally across various healthcare settings and cultural contexts, suggesting the need for multicenter studies to confirm these insights. The strict inclusion and exclusion criteria, focusing solely on medication processes managed by nursing staff, limit the scope of our analysis. This selective approach excludes cases managed by other medical professionals, potentially overlooking the broader dynamics of perioperative medication administration and skewing our understanding of medication error rates. Additionally, excluding records with incomplete documentation could introduce selection bias, possibly underestimating the true prevalence of medication errors. By concentrating only on the nursing aspect of medication administration, our study may not capture all factors that influence medication safety, including systemic, interprofessional, and organizational elements. Moreover, adhering strictly to medication practicality and generic drug consistency, as mandated by regulatory standards, may not fully account for the nuances of individual patient responses and the specific contexts of surgical care. These limitations highlight the need for future research to adopt more inclusive and diverse methodological approaches, extend beyond a single healthcare setting, and involve a wider range of healthcare professionals to fully capture the complexities of medication safety in perioperative care.

In our study, the increased error rate observed in the elderly patient group raises important considerations. Elderly patients often have complex medical needs and polypharmacy, which can increase the likelihood of medication errors due to factors like altered pharmacokinetics and pharmacodynamics associated with aging. This complexity necessitates meticulous attention to medication management, which should be emphasized in nursing education and training. Furthermore, our findings underscore the critical role of practical applications in educational settings. By integrating more hands-on experiences into the curriculum, nurses can develop a deeper understanding and proficiency in managing these complex scenarios, ultimately reducing the risk of errors. As technology continues to advance, its incorporation into both practice and education offers significant potential to enhance learning outcomes and improve patient safety, particularly through the use of simulation-based training. Such training can replicate real-life scenarios, allowing nurses to hone their skills in a controlled, yet realistic environment. Therefore, we advocate for a substantial increase in practical applications during training, which, alongside technological advancements, will likely lead to a notable decrease in medication errors.

In conclusion, this study contributes to the growing body of evidence on perioperative medication errors, highlighting the complex interplay of patient, medication, and healthcare professional factors. Our findings suggest that targeted interventions, focusing on older patients, specific types of medications and routes of administration, and the professional development of nursing staff, could significantly reduce the risk of medication errors. Further research is warranted to explore the efficacy of such interventions in diverse healthcare settings.

## Acknowledgments

We would like to express our sincere gratitude to all individuals who contributed to the completion of this study.

## Author contributions

**Conceptualization:** Lu Mo, Zhongxun Wu.

**Data curation:** Lu Mo, Zhongxun Wu.

**Formal analysis:** Lu Mo, Zhongxun Wu.

**Funding acquisition:** Lu Mo.

**Investigation:** Zhongxun Wu.

**Methodology:** Zhongxun Wu.

**Software:** Zhongxun Wu.

**Writing – original draft:** Zhongxun Wu.

**Writing – review & editing:** Zhongxun Wu.

## References

[1] Ye J. Patient safety of perioperative medication through the lens of digital health and artificial intelligence. JMIR Perioper Med. 2023;6:e34453. 37256663 10.2196/34453 PMC10267793

[2] Cierniak KH Gaunt MJ Grissinger M. Perioperative medication errors: uncovering risk from behind the drapes. Penn Patient Saf Advis. 2018;15:22–38.

[3] Joint Commission. National patient safety goals effective July 2020 for the hospital program. Nat Patient Safet Goals. 2020:1–14.

[4] Rodziewicz TL Houseman B Hipskind JE. Medical error reduction and prevention. In StatPearls. StatPearls Publishing. 2018.

[5] Coelho F Furtado L Mendonça N. Interventions to minimize medication error by nurses in intensive care: a scoping review protocol. Nurs Rep. 2023;13:1040–50. 37606459 10.3390/nursrep13030091 PMC10443247

[6] Wondmieneh A Alemu W Tadele N Demis A. Medication administration errors and contributing factors among nurses: a cross sectional study in tertiary hospitals, Addis Ababa, Ethiopia. BMC Nurs. 2020;19:4. 31956293 10.1186/s12912-020-0397-0 PMC6958590

[7] Wall J Dhesi J Snowden C Swart M. Perioperative medicine. Future Healthc J. 2022;9:138–43. 35928202 10.7861/fhj.2022-0051 PMC9345229

[8] Gopal DP Chetty U O’Donnell P Gajria C Blackadder-Weinstein J. Implicit bias in healthcare: clinical practice, research and decision making. Future Healthc J. 2021;8:40–8. 33791459 10.7861/fhj.2020-0233 PMC8004354

[9] Thirsk LM Panchuk JT Stahlke S Hagtvedt R. Cognitive and implicit biases in nurses’ judgment and decision-making: a scoping review. Int J Nurs Stud. 2022;133:104284. 35696809 10.1016/j.ijnurstu.2022.104284

[10] Abdel-Latif MM. Knowledge of healthcare professionals about medication errors in hospitals. J Basic Clin Pharm. 2016;7:87–92. 27330261 10.4103/0976-0105.183264 PMC4910473

[11] Shitu Z Aung MMT Tuan Kamauzaman TH Ab Rahman AF. Prevalence and characteristics of medication errors at an emergency department of a teaching hospital in Malaysia. BMC Health Serv Res. 2020;20:56. 31969138 10.1186/s12913-020-4921-4 PMC6977341

[12] Naseralallah L Stewart D Price M Paudyal V. Prevalence, contributing factors, and interventions to reduce medication errors in outpatient and ambulatory settings: a systematic review. Int J Clin Pharm. 2023;45:1359–77. 37682400 10.1007/s11096-023-01626-5 PMC10682158

[13] Kerari A Innab A. The influence of nurses’ characteristics on medication administration errors: an integrative review. SAGE Open Nurs. 2021;7:23779608211025802. 34222653 10.1177/23779608211025802 PMC8223601

[14] Tsegaye D Alem G Tessema Z Alebachew W. Medication administration errors and associated factors among nurses. Int J Gen Med. 2020;13:1621–32. 33376387 10.2147/IJGM.S289452 PMC7764714

[15] Rasool MF Rehman AU Imran I. Risk factors associated with medication errors among patients suffering from chronic disorders. Front Public Health. 2020;8:531038. 33330300 10.3389/fpubh.2020.531038 PMC7710866

[16] Sears K Beigi P Niyyati SS Egan R. Patient-related risk factors for the occurrence of patient-reported medication errors in one community pharmacy: a local perspective. J Pharm Technol. 2016;32:3–8. 34860958 10.1177/8755122515596539 PMC5998408

[17] Nanji KC Patel A Shaikh S Seger DL Bates DW. Evaluation of perioperative medication errors and adverse drug events. Anesthesiology. 2016;124:25–34. 26501385 10.1097/ALN.0000000000000904 PMC4681677

[18] Assiri GA Alkhenizan AH Al-Khani SA Grant LM Sheikh A. Investigating the epidemiology of medication errors in adults in community care settings. A retrospective cohort study in central Saudi Arabia. Saudi Med J. 2019;40:158–67. 30723861 10.15537/smj.2019.2.23933 PMC6402461

[19] Abbasi S Rashid S Khan FA. A retrospective analysis of peri-operative medication errors from a low-middle income country. Sci Rep. 2022;12:12404. 35858974 10.1038/s41598-022-16479-7 PMC9300725

[20] Stipp MM Deng H Kong K Moore S Hickman RL Jr Nanji KC. Medication safety in the perioperative setting: a comparison of methods for detecting medication errors and adverse medication events. Medicine (Baltimore). 2022;101:e31432. 36343025 10.1097/MD.0000000000031432 PMC9646678

[21] Wang B Hu L Hu X Han D Wu J. Exploring perioperative risk factors for poor recovery of postoperative gastrointestinal function following gynecological surgery: a retrospective cohort study. Heliyon. 2023;10:e23706. 38205292 10.1016/j.heliyon.2023.e23706 PMC10776945

